# Effects of systemic inflammation and frailty on survival in elderly cancer patients: Results from the INSCOC study

**DOI:** 10.3389/fimmu.2023.936904

**Published:** 2023-02-20

**Authors:** Qi Zhang, Ziwen Wang, Mengmeng Song, Tong Liu, Jiashan Ding, Li Deng, Xi Zhang, Liang Qian, Yizhong Ge, Hailun Xie, Guotian Ruan, Chunhua Song, Qinghua Yao, Hongxia Xu, Haixing Ju, Hanping Shi

**Affiliations:** ^1^ Department of Colorectal Surgery, The Cancer Hospital of the University of Chinese Academy of Sciences (Zhejiang Cancer Hospital) Hangzhou, Zhejiang, China; ^2^ Key Laboratory of Cancer Food for Special Medical Purposes (FSMP) for State Market Regulation, Beijing Shijitan Hospital, Beijing, China; ^3^ Beijing International Science and Technology Cooperation Base for Cancer Metabolism and Nutrition, Beijing Shijitan Hospital, Beijing, China; ^4^ Integrated Traditional Chinese and Western Medicine Oncology Laboratory, Key Laboratory of Traditional Chinese Medicine of Zhejiang Province, Hangzhou, Zhejiang, China; ^5^ Department of Cardiology, Geriatric Cardiovascular Disease Research and Treatment Center, the 82nd Group Army Hospital of PLA (252 Hospital of PLA), Baoding, Hebei, China; ^6^ Department of Gastrointestinal Surgery, Beijing Shijitan Hospital, Capital Medical University, Beijing, China; ^7^ Department of Clinical Nutrition, Beijing Shijitan Hospital, Capital Medical University, Beijing, China; ^8^ Department of Obstetrics and Gynecology, the First Affiliated Hospital of Nanchang University, Nanchang, Jiangxi, China; ^9^ Department of Obstetrics and Gynecology, Hangzhou Women’s Hospital, Hangzhou Maternal and Child Health Hospital, Hangzhou First People’s Hospital Qianjiang, Hangzhou, China; ^10^ Department of Oncology, The Second Affiliated Hospital and Yuying Children's Hospital of Wenzhou Medical University, Wenzhou, China; ^11^ Department of Epidemiology, College of Public Health, Zhengzhou University, Zhengzhou, Henan, China; ^12^ Department of Integrated Chinese and Western Medicine, Cancer Hospital of University of Chinese Academy of Science, Institute of Cancer Research and Basic Medical Sciences of Chinese Academy of Sciences, Zhejiang Cancer Hospital, Hangzhou, China; ^13^ Department of Clinical Nutrition, Daping Hospital, Army Medical University, Chongqing, China

**Keywords:** frailty, systematic inflammation, neutrophil to lymphocyte ratio, cancer, elderly

## Abstract

**Background:**

Frailty and systemic inflammation are parameters, which are easy to evaluate, can be used to predict disease outcomes, and are potentially modifiable. The combination of frailty and inflammation-based data may help identify elderly cancer patients predisposed to adverse clinical outcomes. The aim of this study was to examine the association of systemic inflammation and frailty at admission, and to determine whether these risk factors interact and may predict the survival of elderly cancer patients.

**Methods:**

A prospective Investigation on Nutrition Status and Clinical Outcome of Common Cancers (INSCOC) with 5,106 elderly cancer patients admitted from 2013 through 2020 was included in this study. The primary marker of inflammation was the neutrophil-to-lymphocyte ratio (NLR), with the reference group having NLR<3, which indicated no inflammation. Frailty was assessed using the FRAIL scale, and patients with≥3 positives out of a total of five components were assumed to be frail. The primary outcome was all-cause mortality. We classified participants according to the presence (or absence) of frailty and high inflammation and assessed their association with overall survival using the Cox proportional hazards models adjusted for demographic, tumor, and treatment factors.

**Results:**

Among the 5,106 patients enrolled in the study, 3396 individuals (66.51%) were male and the mean( ± SD) age at diagnosis was 70.92( ± 5.34). Over a median of 33.5 months follow-up, we observed 2,315 deaths. Increasing NLR was associated with frailty (compared with NLR<3, odds ratio=1.23, 95%CI=1.08-1.41 for NLR≥3). An NLR≥3 and frailty independently predicted the overall survival [hazard ratio(HR)=1.35, 95%CI=1.24-1.47 and HR=1.38, 95%CI=1.25-1.52, respectively). Patients with both frailty and NLR≥3 had the lowest overall survival(HR=1.83, 95%CI=1.59-2.04) than patients with no risk factors. The mortality rate increased with the presence of the frailty components.

**Conclusions:**

Systemic inflammation was positively associated with frailty. Frail elderly cancer patients with elevated systemic inflammation had low survival rate.

## Highlights

- **Question**: Is systemic inflammation associated with frailty, and are these 2 risk factors combined associated with survival in elderly patients with cancer.
- **Findings**: In a cohort of 5,106 elderly patients with cancer, elevated neutrophil to lymphocyte ratio(NLR) at admission was associated with frailty; patients with both frailty and NLR≥3 (vs neither) had much worse of overall survival. The hazard of death increased with the number of frailty components present.
- **Meaning**: Frailty and inflammation predicted worse prognosis in elderly patients with cancer regardless of ageing and disease itself. Because these 2 biomarkers are commonly collected and potentially modifiable, they have high potential for clinical use in prognostication and possibly in guiding intervention.

## Introduction

Identifying the elderly cancer patients at higher risks of adverse treatment outcomes and mortality is a clinical priority. Two novel clinical prognostic indicators receiving increasing attention across different cancer types include frailty and elevated neutrophil-to-lymphocyte ratio (NLR, a marker of systemic inflammation) (1). Frailty is a state of vulnerability, and it is recognized as a strong predictor of adverse clinical outcomes (2). However, which frailty parameter is optimal for defining the high chance of adverse clinical outcomes in cancer patients is still unclear (more than 70 different parameters exist) (3). The most extensively studied and used tool to identify frailty in oncology is the comprehensive geriatric assessment (CGA) (4). Administering a full CGA can take hours to complete and is often impractical. The FRAIL scale is a validated screening tool developed by the advisory panel of the International Academy of Nutrition and Aging (IANA). However, the FRAIL scale has not been explored in elderly patients with cancer (5).

Low-grade inflammation plays a critical role in the development of cancer as well as its prognosis (6). In this vein, values of NLR and related blood markers [such as platelet-to-lymphocyte ratio (PLR), lymphocyte-C-reactive protein ratio (LCR), and albumin] are commonly used to predict the survival in cancer patients (7). The direct link between elevated systemic inflammatory markers and frailty has not been established yet (8). The inflammation mediators may contribute to the development and progression of sarcopenia, which is prevalent in older patients and is a hallmark of frailty (9). From this perspective, a hypothesis to explain the link between inflammation and frailty is that inflammation (such as IL-6, IL-1α, TNF-α) is associated with reduced synthesis and activity of IGF1, a growth factor that is essential for muscle regeneration and maintenance of muscle integrity (10). Not only that, inflammation impairs endothelial reactivity and muscle perfusion, interfering with the uptake of long branched-chain amino acids that are essential for muscle energetics and protein anabolism (11)Additionally, recent studies have shown that obesity contributes to ageing-related pathologies, such as elevated inflammation, insulin resistance, and cellular senescence, which in turn likely to contribute to an increased risk of frailty (12).

To date, the majority of the research has been focused on examining the correlation between systemic inflammation and frailty (13). To our knowledge, no prior study has examined the combined effects of systemic inflammation and frailty on the survival of elderly cancer patients. It is important because inflammation status may help identify high-risk subgroup of frailty patients. In this study, we examined the association of NLR with frailty. Subsequently, we assessed whether four categories defined by the presence/absence of frailty and presence/absence of systemic inflammation were associated with survival.

## Methods

### Study population

This study includes an “Investigation on Nutrition Status and its Clinical Outcome of Common Cancers (INSCOC)” cohort, which has been described in detail previously (6, 14, 15). The cohort was registered at http://www.chictr.org.cn (registration number ChiCTR1800020329). In brief, the study included more than 50,000 patients who were diagnosed with cancer between 2013 and 2020 in China. The cohort has been followed since 2013. Informed consent was obtained from all patients before participation, and institutional review boards at all sites approved the study. For this study, we restricted the sample to those older than 65 years. Patients with clinical evidence of acute infection were also excluded from our study. For the analysis of different biomarkers, the sample size varied according to the number of missing data for the biomarkers or covariates. Patients included and excluded due to insufficient data were similar in sex, age, BMI, primary tumor location, and tumor stage (data not show). All experimental protocols were approved by ethical licensing committee of army medical center of Daping hospital. All methods were carried out in accordance with Helsinki.

### Markers of systemic inflammation

The primary mark of systemic inflammation was NLR from laboratory values obtained as part of routine blood tests (with all measurements being made before the treatment interventions of surgery, chemotherapy, radiotherapy or other treatments). We categorized the NLR value using standard cutoffs to define the normal condition (<3) and that of high inflammatory state (≥3) (16). The data of the secondary biomarkers of systemic inflammation (PLR; LCR and albumin) were collected at the same time point as that of the primary biomarker. We categorized available markers using clinically relevant cut offs (PLR: <150, 150 to 300, ≥300; LCR: <3000 and ≥3000; albumin: <3.5 g/L and ≥3.5 g/L, respectively) (7).

### Frailty categories

We defined frailty using the FRAIL scale, whereby individuals with positivity for three or more parameters of a five-component system consisting of: fatigue, resistance, ambulation, illness, and loss of weight, respectively, were designated as frail (17)(details are presented in **Supplementary Table 1**). However, some of these items were adapted to fit the INSCOC data available. We categorized patients according to presence/absence of frailty and presence/absence of systemic inflammation (frailty only, systemic inflammation only, both or neither) first. To evaluate the dose-response relationship of frailty and survival, we summarized all the frailty components present (the number of risk components ranging from 0 to 5). A total of 14 patients scored 5, while 845 patients scored 4 on the FRAIL scale. The groups were grouped because of small frequencies.

### End points and other covariate data

The outcomes studied included all-cause mortality at any time point after enrollment in the study and mortality after 30 days of hospitalization. For overall survival, we calculated the time from the date of the first admission to the data of death or the pre-selected deadline of December 30, 2020. Follow-up information on survival data of patients was mainly collected by telephone or from annual outpatient reexamination schedules.

Potential confounding variables included in this study were based on the previous literature (6). The patient-based demographic information (sex, age, smoking habits, alcohol consumption), medical history (tumor type or stage), and treatment history (surgery, chemotherapy, radiotherapy and nutrition intervention) were compiled from the electronic medical record system, Information about the place of residence, educational qualification, patient-generated subjective global assessment (PG-SGA) for nutritional status, EORTC Core Quality of Life questionnaire (EORTC QLQ‐C30) for quality of life, and the Eastern Cooperative Oncology Group (ECOG) for performance status was collected by trained staff in a personal interview session using a questionnaire. Physical measurements were recorded using calibrated instruments following standard procedures. Briefly, body weight and height are measured in light indoor clothing without shoes, to the nearest 0.1 kg and0.1 cm, respectively. The hand grip strength (HGS) is measured by an electronic hand grip dynamometer (CAMRY, Model EH101, Guagndong, China).

### Statistical analysis

Descriptive statistical analyses were performed to present frailty among patients and to compare characteristics between patients with or without systemic inflammation. Results were obtained as mean values (mean ± SD) or absolute numbers (%) as applicable and appropriate. We conducted logistic regression for categorical NLR (<3 and ≥3) as a predictor of frailty (presence or absence), and presented the data using odds ratios (ORs) with 95% CI. The Pearson correlation coefficient and kappa coefficient were used to describe the association between each systemic information marker (NLR, PLR, LCR, and albumin). The association of four categories defined by frailty with or without the presence of systemic inflammation and overall survival was examined and the Kaplan-Meier curves were plotted. The Cox regression models were used to calculate hazard ratios (HRs) and 95% CIs, respectively. The models were adjusted for age, gender, BMI, smoking habits, alcohol consumption, tumor location, tumor stage, chemotherapy, radiotherapy, surgery, nutrition intervention, ECOG, PG-SGA, place of residence, educational qualification, HGS, and EORTC QLQ‐C30, respectively. Logistic regression models were used to estimate the association of frailty and systemic inflammation with that of parameter of 30-day mortality. The presence of multiplicative interaction between frailty and NLR was also evaluated. We accounted for potential reverse causality by excluding patients dying within the first six months or ECOG less than one score after baseline. We also examined individual and mutually adjusted association of individual frailty components with that of overall survival. The C-statistic was used to assess the discrimination of the model. The two-tailed P value of <0.05 was considered significant. We used R software (version 4.0.1, https://www.r-project.org/) for all statistical analyses.

## Results

### Population characteristics

**Table 1** shows patient characteristics (n=5106) and overall survival, which has been categorized based on the frailty parameters. Of all the patients, 66.51% were males and the mean ( ± SD) age at admission was 70.92 years ( ± 5.34), mean ECOG performance status was 1.08 ( ± 0.75), with 66.43% having received surgery, 14.96% having received radiotherapy, and 57.17% patients having received chemotherapy. Advanced stages of cancer (III/IV) were detected in 3524 patients (69.02%). The most common tumor type was that of the digestive system (51.72%), followed by that of the lungs (27.97%). Patients without frailty had greater HGS than frail patients. The prevalence of frailty was 45.05%. The absence of frailty (fewer than three frailty risk factors) and low systemic inflammation was observed in younger patients (70.32 ± 5.18 years) with higher BMI (23.16 ± 3.32 kg m-2) than other groups. Among frail patients with high systemic inflammation, 23.18% had four or more abnormal frailty-based risk factors, while only 11.79% of the frail patients exhibited low systemic inflammation (**Supplementary Table 2**).

**Table 1:** Characteristics of elderly Patients With Cancer, Overall and by systemic inflammation, frailty.

Characteristic	All	NLR<3		*P*-value	NLR≥3		*P*-value
		No frailty	Frailty		No frailty	Frailty	
	n=5106	n=1779	n=1071		n=1027	n=1229	
Gender				<0.001			0.001
Male	3396 (66.51%)	1166 (65.54%)	629 (58.73%)		765 (74.49%)	836 (68.02%)	
Female	1710 (33.49%)	613 (34.46%)	442 (41.27%)		262 (25.51%)	393 (31.98%)	
Age, mean (SD), years	70.92 (5.34)	70.32 (5.18)	71.15 (5.30)	<0.001	70.63 (5.26)	71.81 (5.55)	<0.001
BMI, mean (SD), kg/m^2^	22.26 (3.46)	23.16 (3.32)	21.84 (3.53)	<0.001	22.43 (3.29)	21.19 (3.38)	<0.001
Smoking, yes	3465 (67.86%)	1216 (68.35%)	769 (71.80%)	0.058	666 (64.85%)	814 (66.23%)	0.519
Alcohol, yes	4060 (79.51%)	1408 (79.15%)	893 (83.38%)	0.006	788 (76.73%)	971 (79.01%)	0.211
Tumor types				<0.001			0.001
Lung cancer	1428 (27.97%)	425 (23.89%)	308 (28.76%)		300 (29.21%)	395 (32.14%)	
Digestive cancer^a^	1491 (29.20%)	457 (25.69%)	365 (34.08%)		280 (27.26%)	389 (31.65%)	
Colorectal cancer	1147 (22.46%)	415 (23.33%)	210 (19.61%)		249 (24.25%)	273 (22.21%)	
Others	1040 (20.37%)	482 (27.09%)	188 (17.55%)		198 (19.28%)	172 (14.00%)	
Tumor stage				<0.001			<0.001
I	507 (9.93%)	219 (12.31%)	90 (8.40%)		118 (11.49%)	80 (6.51%)	
II	1075 (21.05%)	457 (25.69%)	204 (19.05%)		228 (22.20%)	186 (15.13%)	
III	1330 (26.05%)	527 (29.62%)	276 (25.77%)		274 (26.68%)	253 (20.59%)	
IV	2194 (42.97%)	576 (32.38%)	501 (46.78%)		407 (39.63%)	710 (57.77%)	
Surgery, yes	3392 (66.43%)	1289 (72.46%)	692 (64.61%)	<0.001	689 (67.09%)	722 (58.75%)	<0.001
Radiotherapy, yes	764 (14.96%)	240 (13.49%)	146 (13.63%)	0.960	167 (16.26%)	211 (17.17%)	0.604
Chemotherapy, yes	2919 (57.17%)	1011 (56.83%)	696 (64.99%)	<0.001	539 (52.48%)	673 (54.76%)	0.299
Nutrition intervention, yes	1928 (37.76%)	551 (30.97%)	397 (37.07%)	0.001	374 (36.42%)	606 (49.31%)	<0.001
ECOG, mean (SD)	1.08 (0.75)	0.76 (0.56)	1.23 (0.66)	<0.001	0.89 (0.62)	1.57 (0.89)	<0.001
PG-SGA, mean (SD)	7.14 (4.95)	4.82 (3.51)	8.48 (4.91)	<0.001	5.75 (3.85)	10.50 (5.33)	<0.001
Place of Residence				0.095			0.348
Urban	2521 (49.37%)	886 (49.80%)	498 (46.50%)		506 (49.27%)	631 (51.34%)	
Rural	2585 (50.63%)	893 (50.20%)	573 (53.50%)		521 (50.73%)	598 (48.66%)	
Educational attainment				0.046			0.052
Below primary education	604 (11.83%)	186 (10.46%)	142 (13.26%)		107 (10.42%)	169 (13.75%)	
Primary education	3844 (75.28%)	1371 (77.07%)	786 (73.39%)		781 (76.05%)	906 (73.72%)	
Upper secondary education	658 (12.89%)	222 (12.48%)	143 (13.35%)		139 (13.53%)	154 (12.53%)	
HGS, mean (SD), kg	22.48 (8.98)	24.69 (9.03)	20.66 (8.46)	<0.001	24.25 (8.66)	19.40 (8.39)	<0.001
EORTC QLQ-C30, mean (SD)	39.46 (4.83)	39.70 (3.90)	39.23 (5.42)	0.014	39.57 (4.25)	39.21 (5.83)	0.089
NLR, median (quartiles)	1.87 (1.40;2.37)	1.85 (1.40;2.37)	1.91 (1.39;2.38)	0.447	4.52 (3.61;6.67)	5.40 (3.92;8.20)	<0.001

Data presented as No.(%) unless otherwise noted. Percentages may not add to 100% because of rounding.

BMI, body mass index; SD, standard deviation; ECOG, Eastern Cooperative Oncology Group; PG-SGA, patient-generated subjective global assessment; HGS: hand grip strength; EORTC QLQ‐C30: European Organization for Research and Treatment of Cancer Quality of Life Questionnaire.

a Digestive cancer except colorectal cancer

### Association of systemic inflammation and frailty

A higher NLR was positively associated with the parameters of frailty independent of age, gender, BMI, tumor location, tumor stage, and ECOG, respectively. Compared with NLR of less than 3, the OR for frailty was 1.23 (95% CI,1.08-1.41). Results were consistent across other markers of systemic inflammation, which included high PLR, high LCR, and clinical condition of hypoalbuminemia, which were all associated with high ORs of frailty and severe malnutrition (**Supplementary Table 3**). Furthermore, the association of elevated NLR with increased ORs of frailty was consistent across sexes, tumor types, and different cancer stages (**Supplementary Table 4**, no evidence of interaction in multivariable analyses). However, the correlation was poor between the inflammatory markers (**Supplementary Table 5**).

### Outcome of elevated inflammation and frailty

During a median follow-up period of 33.5 months (range: 9 days to 8 years), we observed 2,315 deaths. Univariable analyses for risk factors for overall survival have been presented in **Supplementary Table 6**. The incidence of death due to high frailty and NLR (events per 1000 person-years) were 332.87 and 172.08, respectively. The numbers of risk factors which increased with age have been presented in **Supplementary Figure 2**, which illustrates that frailty and systemic inflammation were common in older patients.

**Figure 1:**
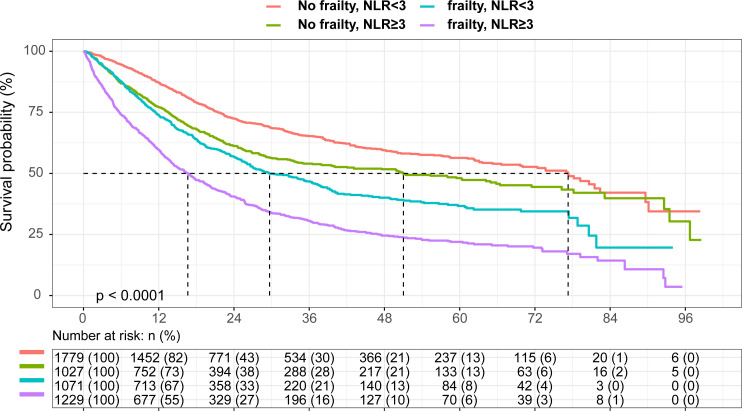
Kaplan-Meier curve According to Neutrophil to Lymphocyte Ratio and Frailty Present in Elderly Patients with Cancer.

As observed in **Figure 1**, patients with both frailty and NLR≥3 had the shortest survival, whereas patients without frailty and NLR<3 survived the longest (log-rank P < 0.001). Survival probabilities for frailty and NLR≥3 have been shown in **Supplementary Figure 3**. **Table 2** shows multivariable-adjusted associations of our main exposure (each risk factor and four categories defined by frailty and NLR) with overall survival. Frailty and NLR≥3 independently predicted the overall survival showing an HR= 1.35 (95% CI,1.24-1.47) for NLR≥3, while for frailty the HR was 1.38 (95% CI, 1.25-1.52). Patients with both frailty and high NLR were estimated to have a much shorter overall survival (HR:1.80, 95% CI, 1.59-2.04) than patients with neither risk factors. Similarly, cumulative incidence curves for death suggested greater mortality among patients with frailty or a normal NLR (**Supplementary Figure 4**).

**Table 2:** Systemic Inflammation (NLR), frailty, and survival in elderly patients with cancer.

	Model^a^	Model^b^	Model^c^
	HR(95%CI)	HR(95%CI)	HR(95%CI)s
Frailty			
No	Ref.	Ref.	Ref.
Yes	2.06 (1.90-2.24)***	1.93 (1.78-2.11)***	1.38 (1.25-1.52)***
NLR			
<3	Ref.	Ref.	Ref.
≥3	1.7 (1.57-1.84)***	1.56 (1.43-1.69)***	1.35 (1.24-1.47)***
Frailty and NLR			
No, <3	Ref.	Ref.	Ref.
No, ≥3	1.44 (1.27-1.63)***	1.34 (1.18-1.52)***	1.27 (1.12-1.45)***
Yes, <3	1.81 (1.60-2.04)***	1.72 (1.53-1.95)***	1.30 (1.14-1.48)***
Yes, ≥3	2.96 (2.65-3.29)***	2.63 (2.36-2.94)***	1.80 (1.59-2.04)***

Data presented as hazard ratio (95% CI). ***P-value <0.001.

a Cox proportional hazards models without adjust;

b Cox proportional hazards models adjust for age, gender, body mass index;

c Cox proportional hazards models adjust for age, gender, body mass index, smoking, alcohol, tumor location, tumor stage, chemotherapy, radiotherapy, surgery, nutrition intervention, ECOG, PG-SGA, Place of Residence, Educational attainment, hand grip strength, EORTC QLQ‐C30.

### “FRAIL” scale components and the mortality

**Figure 2:**
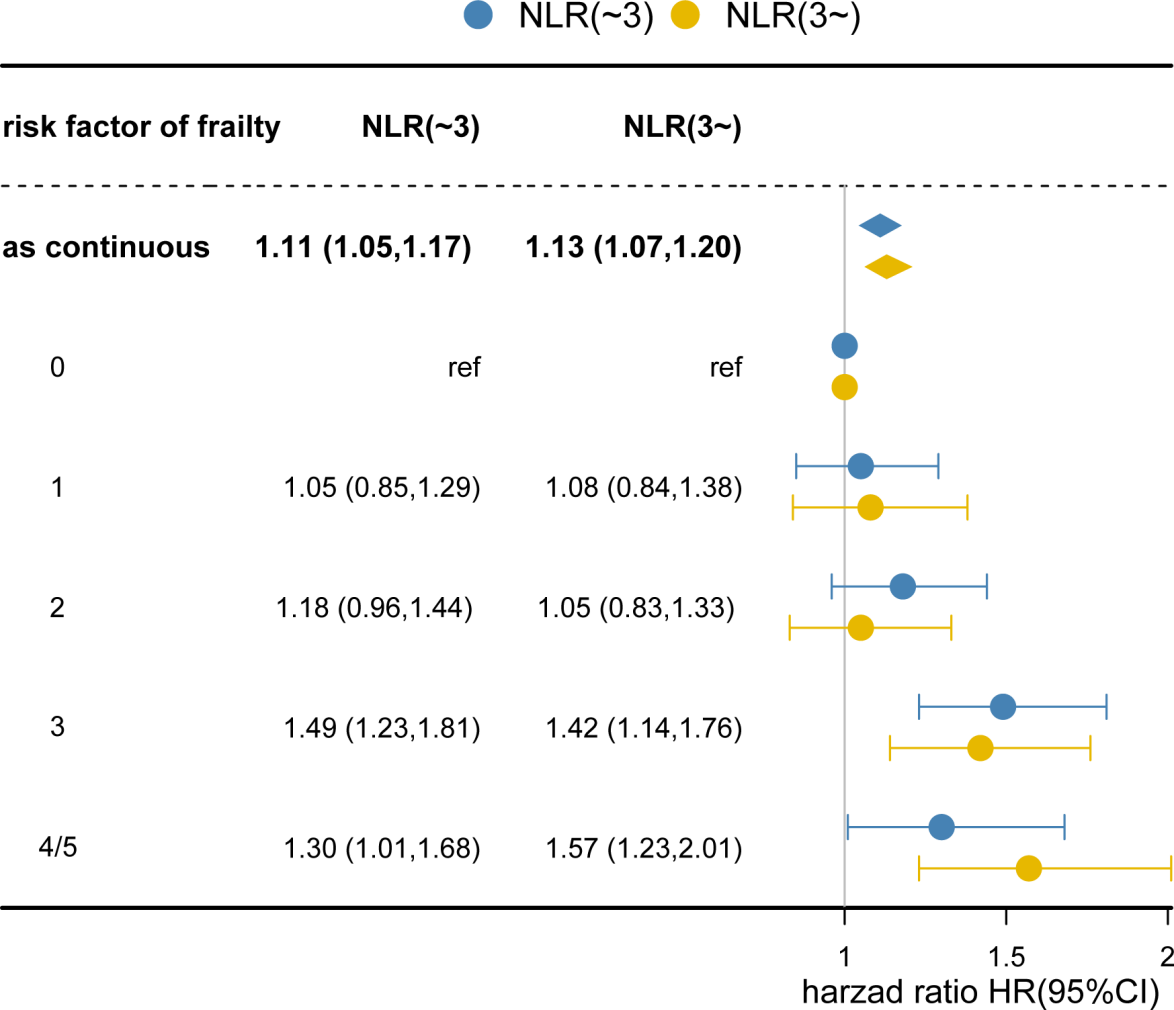
Degree of Failty at Admission and Overall Survival. Each additional frailty component present at admission increased hazard of death by 11% (hazard ratio [HR], 1.11; 95% CI, 1.05-1.17; P <0.001) for NLR<3 and 13% (HR: 1.13; 95% CI, 1.07-1.20; P<0.001) for NLR≥3. Cox regression models adjusted for age, gender, body mass index, smoking, alcohol, tumor location, tumor stage, chemotherapy, radiotherapy, surgery, nutrition intervention, ECOG, PG-SGA, Place of Residence, Educational attainment, hand grip strength, EORTC QLQ‐C30. Number of risk factors indicates the number of abnormal frailty components present at admission. The reference group had 0 frailty risk factors.

**Table 3** and **Supplementary Figure 6** summarize the frailty components and correlate them with survival. When all frailty risk factors were mutually adjusted, only ambulation (HR: 1.21, 95% CI, 1.06-1.37) and fatigue (HR: 1.25, 95% CI, 1.12-1.38) remained associated with overall survival. Since the majority of patients had at least one frailty risk factor, we evaluated the dose-response relationship of severity of the risk of frailty with survival (**Supplementary Figure 7**). Overall survival decreased with the presence of additional frailty risk factors independent of the NLR category (**Figure 2**). Dose-response trends for overall survival were significant (HR:1.11, 95% CI,1.05-1.17 and HR: 1.13, 95% CI, 1.07-1.20, respectively, for each additional frailty risk factor). There was no difference in survival when comparing patients with zero, one or two risk factors. For patients positive with three risk factors and those with four or more risk factors the overall survival was significantly decreased.

**Table 3:** Associations of Frailty and Components With Overall Survival.

Risk Factor	n	Crude HR (95%CI)	*P*-value	Adjusted for model c	*P*-value	Mutually adjusted	*P*-value
Resistance		1.74 (1.59-1.90)	<0.001	1.20 (1.09-1.32)	<0.001	0.98 (0.86-1.11)	0.730
Ambulation		1.86 (1.71-2.04)	<0.001	1.30 (1.18-1.43)	<0.001	1.21 (1.06-1.37)	0.004
Fatigue		1.94 (1.79-2.12)	<0.001	1.33 (1.21-1.46)	<0.001	1.25 (1.12-1.38)	<0.001
Illness		1.30 (0.96-1.76)	0.095	1.24 (0.91-1.69)	0.175	1.19 (0.87-1.62)	0.275
Lost		1.52 (1.39-1.65)	<0.001	1.02 (0.92-1.14)	0.657	1.04 (0.94-1.16)	0.445

Model c: Cox proportional hazards models adjust for Adjusted by age, gender, body mass index, smoking, alcohol, tumor location, tumor stage, chemotherapy, radiotherapy, surgery, nutrition intervention, ECOG, PG-SGA, Place of Residence, Educational attainment, hand grip strength, EORTC QLQ‐C30.

In the mutually adjusted row, each frailty risk factor is adjusted for all others in the row as well as for the covariates listed above.

### Sensitivity analyses

There was no statistical evidence that sex, tumor type, tumor stage, treatment method, and nutritional status modified the association of frailty with NLR≥3 with overall survival (**Supplementary Table 7**). Sensitivity analyses did not subsequently alter the afore-mentioned conclusion (**Supplementary Table 8**), which accounted for ECOG ≤ 1, excluding patients dying within 6 months. In the nutrition treatment subgroup, the frail patients with NLR≥3 had significantly worse outcomes (**Supplementary Figure 5**). Indeed, the afore-mentioned group (positive for frailty with NLR≥3) had almost three times adjusted odds of 30-day mortality than those who were negative for any of the risk factors (OR: 2.80, 95% CI, 1.24-6.29, **Supplementary Table 9**). Most importantly, the addition of frailty and NLR showed a clinically meaningful improvement of 3.7% in the concordance index (C-index).

## Discussion

In this study, 5,106 elderly patients with cancer were enrolled in whom an increased systemic inflammation was found to be associated with frailty. The prevalence of frailty in older cancer patients is high, with a median estimate of 42% being reported previously (2), which is consistent with our result. The frailty independently correlated with increased levels of inflammatory markers and lower albumin levels. The combination of frailty and systemic inflammation was associated with lowest overall survival, 30-day mortality, and quality of life. The hazard of death increased in a dose-response manner with additional frailty risk factors, suggesting that the presence of each frailty factor incrementally worsened the outcome.

Frailty and systemic inflammation can be easily assessed in the clinical setting and each of them represents an independently powerful prognostic indicator in elderly patients with cancer (18, 19). However, most studies addressed them individually in elderly patients with cancer (20). A comprehensive list of inflammatory markers that are associated with frailty or aging has not yet been compiled (21). NLR is the most studied cellular marker of inflammation and is consistently reported to be associated with frailty in multiple studies. Our study suggests that elevated NLR and other aberrations in inflammatory markers were present in nearly half of patients during their initial admission in the clinic, and these were often associated with frailty, and can predict survival accurately. Furthermore, we observed similar results independent of the sex, primary tumor type, and stage in the patients. The importance of inflammation in frailty has consistently been shown in elderly patients with cancer (13, 22).

In elderly patients with cancer, factors such as aging, the type of tumor and treatment can also contribute to chronic inflammation (23). Chronic inflammation can actively contribute to accelerated frailty (24). For example, in early breast cancer patients, inflammatory blood biomarkers are positively correlated with progressive aging and deteriorating frailty status (25, 26). Several studies have suggested that elevation in the levels of pro-inflammatory cytokines (e.g. TNF-α, IL-6), may contribute to the increase in monocyte or neutrophil count, and that reduced lymphocyte counts are often associated with frailty syndrome (27, 28). These inflammatory markers involve both the innate and adaptive components of the immune system and could be a hallmark of immunosenescence which is often associated with frailty (29, 30). In our analyses, we adjusted for several factors that might influence inflammatory markers, including age, BMI, treatment type, tumor type and stage, and the association remained significant after correcting for the covariates.

Frailty provides the basis for inflammation since low-grade inflammation caused by tumor has underlying mechanisms to accelerate the progression of frailty (31). As a result, it is not surprising that frailty and inflammation frequently co-exist (13, 32). With respect to survival in cancer patients, few studies have directly compared frailty and inflammation (33). Our findings suggest that the co-occurrence of inflammation and frailty is associated with a high mortality risk, which is consistent with results reported in existing literature (34). Additionally, in our study, the hazard ratios of frailty with inflammation were higher for tumors with relatively long-life expectancies than tumors with higher lethality [HR (95%CI): 2.02 (1.52-2.69) vs. 1.69 (1.36-2.10)]. Similar results were obtained in another study (35). Moreover, smoking, malnutrition, and worsening ECOG-PS were found to be associated with deteriorating frail physiology and mortality in cancer patients (36, 37). However, high mortality risk associated with frailty/inflammation in older patients, even in the absence of these risk factors.

Despite the presence of multiple pathways through which frailty might influence the prognosis of cancer, not all frailty components exhibit adverse relationships. The patients who were feeling fatigued and having trouble taking a long walk showed decreased survival. Enhanced chronic inflammation is related to muscle weakness in the extremities and slow gait speed, which manifests as an indicator of frailty (38). Exercise or lifestyle modification (such as nutrition) can be a promising intervention, with possible benefits such as reduction in the levels of inflammatory markers and resistance to frailty (21). According to the FRAIL scale, patients positive for one or two risk factors can be categorized as pre-frail. Our findings indicate that pre-frailness is not associated with a greater risk of all-cause mortality. However, this is not consistent with previous studies reporting an elevated risk of worst outcomes among those categorized to be in pre-frail condition (39). One potential explanation is that pre-frail patients could became frail or returned to a fit category over time, this should have biased results for the association of pre-frailty and mortality toward the null.

To our knowledge, this is the largest study being reported to date that examines the relationship between markers of systemic inflammation and frailty in elderly patients with cancer, and the only one examining their independent and combined associations with survival. Regarding study limitations, the data used in this study was self-reported, which may be subjective and may have recall bias, even though the prevalence of frailty in this study aligns with the estimates reported in the ELderly CAncer PAtient (ELCAPA) cohort study (37). Moreover, the use of substitutes for unavailable variable-based data may have resulted in classification bias. However, the substitutes used were similar to the original variables. Additionally, we could not assess the causality of the identified associations between frailty and systemic inflammation in this study. Moreover, a longitudinal study is necessary to confirm the results. Finally, although the models were adjusted for a comprehensive list of confounding factors, some of the residual confounding factors or unmeasured confounding factors may also pose obstacles in the generalization of the presented results. For example, medication or genetic data that was not available may have included some factors linking risk factors and mortality. Because of the same reason, we can not to distinguish patients with acute inflammation and chronic inflammation of chronic diseases in this study.

In conclusion, our findings demonstrated that the combination of frailty and inflammation was associated with adverse survival outcomes in elderly cancer patients. Considering that both conditions can be assessed in clinical settings, our findings support the routine assessment of individual frailty and inflammation in elderly cancer patients for guiding the treatment decisions being undertaken. Future research is, however, needed to explore whether intervening on frailty domains can improve functional status, global quality of life, symptom burden or tolerance to cancer therapy.

## Data availability statement

The raw data supporting the conclusions of this article will be made available by the authors, without undue reservation.

## Ethics statement

The studies involving human participants were reviewed and approved by ethics committee of army medical center of PLA. The patients/participants provided their written informed consent to participate in this study.

## Author contributions

HS had full access to all the data in the study and take responsibility for the integrity of the data and the accuracy of the data analysis. Study concept and design: QZ, ZW, JD. Acquisition, analysis, or interpretation of data: QZ, JD. Drafting of the manuscript: QZ. Critical revision of the manuscript for important intellectual content: All authors. Obtained funding: HS. Administrative, technical, or material support: HS, CS, HXX, QY. Supervision: QZ, HS, CS, HLX, QY. Final approval of manuscript: All authors.
