# Supplementary material for: Effects of systemic inflammation and frailty on survival in elderly cancer patients: Results from the INSCOC study
Source: Front Immunol. 2023 Feb 20;14:936904. doi: 10.3389/fimmu.2023.936904 (PMC9986529; doi:10.3389/fimmu.2023.936904)

Supplementary table 1. Frailty definition

Individuals components	Definition used in this manuscript
Fatigue	Self-reported: do you feel weak
Resistance	Self-reported: ability to climb 1 flight of stairs /or do you have any trouble doing strenuous activities, like carrying a heavy shopping bag or a suitcase
Ambulation	Self-reported: ability to walk 1 block /or do you have any trouble taking a long walk
Illness	Greater than 5
Lost of weight	compared with 6 months ago, weight loss more than 5%

Illness: including liver cirrhosis, chronic hepatitis, stroke, COPD, myocardial infarction, diabetes, hypertension, coronary heart disease, anemia, hyperthyroidism, hypothyroidism, chronic pancreatitis, osteoporosis, ulcerative colitis, intestinal Crohn's disease, chronic biliary tract Systemic diseases, chronic kidney disease, tuberculosis, systemic lupus erythematosus

Supplementary table 2. Number of Frailty Characteristics by Level of Systemic Inflammation in Study Participants

**No. of risk factor of frailty**	**Overall N=5106**	**NLR<3 N=2850**	**NLR≥3 N=2256**	***P*-value**
0	1062 (20.80%)	721 (25.30%)	341 (15.12%)	<0.001
1	831 (16.27%)	522 (18.32%)	309 (13.70%)	
2	913 (17.88%)	536 (18.81%)	377 (16.71%)	
3	1441 (28.22%)	735 (25.79%)	706 (31.29%)	
4	845 (16.55%)	331 (11.61%)	514 (22.78%)	
5	14 (0.27%)	5 (0.18%)	9 (0.40%)	

NLR: Neutrophil-to-Lymphocyte Ratio

Supplementary table 3. Association of Additional Markers of Systemic Inflammation With Frailty and severe malnutrition in Elderly Patients with Cancer.

	**Frailty**		**Severe malnutrition***	
	**OR(95%CI)**	***P*-value**	**OR(95%CI)**	***P*-value**
Neutrophil-to-Lymphocyte Ratio				
NLR	Ref		Ref	
NLR	1.23 (1.08-1.41)	0.003	1.38 (1.20,1.59)	< 0.001
Platelet to Lymphocyte Ratio				
PLR: <150	Ref		Ref	
PLR: 150~300	1.03 (0.89-1.18)	0.723	1.11 (0.96,1.29)	0.162
PLR: ≥300	1.34 (1.08-1.65)	0.007	1.4 0(1.13,1.73)	0.002
Lymphocyte-C-reactive Protein Ratio				
LCR: <3000	Ref		Ref	
LCR: ≥3000	0.74 (0.6-0.91)	0.004	0.58 (0.47,0.73)	< 0.001
Albumin				
<3.5g/L	Ref		Ref	
≥3.5g/L	0.73 (0.62-0.85)	< 0.001	0.61 (0.52,0.71)	< 0.001

All models adjusted by age, gender, body mass index, smoking, alcohol, tumor location, tumor stage, chemotherapy, radiotherapy, surgery, nutrition intervention, ECOG, PG-SGA, Place of Residence, Educational attainment, hand grip strength, EORTC QLQ-C30.

*severe malnutrition: PG-SGA>8

Supplementary table 4. Association of Neutrophil-to-Lymphocyte Ratio with Frailty by Gender, Tumor types and Tumor Stage.

	**OR(95%CI)**		***P*-value**
	**NLR<3**	**NLR≥3**	
Gender			
Male	Ref.	1.20 (1.01,1.42)	0.035
Female	Ref.	1.26 (1.00,1.59)	0.052
Tumor types			
Lung cancer	Ref.	1.19 (0.93,1.53)	0.159
Digestive cancer[a]	Ref.	1.16 (0.89,1.49)	0.268
Colorectal cancer	Ref.	1.42 (1.06,1.90)	0.018
Others	Ref.	1.26 (0.9,1.78)	0.180
Tumor stage			
I	Ref.	1.17 (0.73,1.89)	0.510
II	Ref.	1.15 (0.84,1.57)	0.381
III	Ref.	1.25 (0.96,1.64)	0.100
IV	Ref.	1.28 (1.05,1.58)	0.017

All models adjusted by age, gender, body mass index, smoking, alcohol, tumor location, tumor stage, chemotherapy, radiotherapy, surgery, nutrition intervention, ECOG, PG-SGA, Place of Residence, Educational attainment, hand grip strength, EORTC QLQ-C30.

Supplementary table 5. Correlation and Kappa Agreement for Albumin, PLR and LCR compared with NLR.

	**Albumin**	***P*-value**	**PLR**	***P*-value**	**LCR**	***P*-value**
Person	-0.042	0.038	0.498	<0.001	-0.067	0.025
Kappa	***	0.896	***	0.948	***	0.983

PLR: Platelet to Lymphocyte Ratio; LCR: Lymphocyte-C-reactive Protein Ratio

***<0.001

Supplementary table 6. Univariable Cox regression analyses of factors predicting all-cause mortality

	**No. of Event/mean(sd)**	**HR(95%CI)**	***P*-value**
Gender			
Male	1647(71.10%)	Ref.	Ref.
Female	668(28.90%)	0.70(0.64-0.76)	<0.001
Age, years, per sd	71.5(5.50)	1.1(1.09-1.17)	<0.001
BMI			
<18.5	410(17.70%)	Ref.	Ref.
18.5-24	1325(57.20%)	0.69(0.62-0.77)	<0.001
>24	580(25.10%)	0.48(0.42-0.54)	<0.001
Smoking			
No	1521(65.70%)	Ref.	Ref.
Yes	794(34.30%)	1.28(1.18-1.40)	<0.001
alcohol			
No	1791(77.40%)	Ref.	Ref.
Yes	524(22.60%)	1.20(1.08-1.32)	<0.001
Frailty, as continuous	0.57(0.50)	2.06(1.90-2.24)	<0.001
NLR			
<3	1072(46.30%)	Ref.	Ref.
≥3	1243(53.70%)	1.70(1.57-1.84)	<0.001
Tumor types			
Lung cancer	784(33.90%)	Ref.	Ref.
Digestive cancer[a]	802(34.60%)	1.02(0.93-1.13)	0.632
Colorectal cancer	431(18.60%)	0.59(0.52-0.66)	<0.001
Others	298(12.90%)	0.44(0.38-0.50)	<0.001
Tumor stage			
I	99(4.28%)	Ref.	Ref.
II	311(13.40%)	1.52(1.22-1.91)	<0.001
III	550(23.80%)	2.69(2.17-3.33)	<0.001
IV	1355(58.50%)	5.53(4.50-6.78)	<0.001
Surgery			
No	1042(45.00%)	Ref.	Ref.
Yes	1273(55.00%)	0.43(0.40-0.47)	<0.001
Radiotherapy			
No	1924(83.10%)	Ref.	Ref.
Yes	391(16.90%)	1.19(1.07-1.33)	0.002
Chemotherapy			
No	899(38.80%)	Ref.	Ref.
Yes	1416(61.20%)	1.33(1.22-1.44)	<0.001
Nutrition intervention			
No	1407(60.8%)	Ref.	Ref.
Yes	908(39.20%)	1.13(1.04-1.22)	0.005
ECOG, as continuous	1.25(0.83)	1.47(1.40-1.54)	<0.001
PG-SGA			
Absent	536(23.20%)	Ref.	Ref.
Moderate malnutrition	775(33.50%)	1.42(1.27-1.59)	<0.001
Severe malnutrition	1004(43.40%)	2.20(1.98-2.45)	<0.001
Place of Residence			
Urban	1089(47.00%)	Ref.	Ref.

Rural	1226(53.00%)	1.14(1.05-1.24)	0.001
Educational attainment			
Below primary education	286(12.40%)	Ref.	Ref.
Primary education	1754(75.8%)	0.97(0.86-1.10)	0.648
Upper secondary education	275(11.90%)	0.81(0.69-0.96)	0.014
HGS, per kg	21.6(9.05)	0.98(0.98-0.99)	<0.001
EORTC QLQ-C30, as continuous	39.5(5.33)	1.00(0.96-1.05)	0.973

BMI, body mass index; SD, standard deviation; ECOG, Eastern Cooperative Oncology Group; PG-SGA, patient-generated subjective global assessment; HGS: hand grip strength; EORTC QLQ-C30: European Organization for Research and Treatment of Cancer Quality of Life Questionnaire

[a] Digestive cancer except colorectal cancer

Supplementary table 7. Neutrophil to Lymphocyte Ratio (NLR), Frailty , and overall Survival Stratified by Sex, Tumor types, Stage, Treatment, and Nutritional Status.

**Frailty and NLR**	**Model a**	***P*-value**	**Model b**	***P*-value**	**Model c**	***P*-value**
Male						
No, <3	Ref.		Ref.		Ref.	
No, ⩾3	1.33(1.15-1.54)	<0.001	1.29(1.12-1.5)	<0.001	1.22(1.05-1.41)	0.008
Yes, <3	1.83(1.58-2.11)	<0.001	1.69(1.46-1.96)	<0.001	1.27(1.09-1.49)	0.002
Yes, ⩾3	2.84(2.50-3.22)	<0.001	2.55(2.24-2.91)	<0.001	1.76(1.51-2.04)	<0.001
Female						
No, <3	Ref.		Ref.		Ref.	
No, ⩾3	1.56(1.21-2.01)	<0.001	1.47(1.14-1.89)	0.003	1.41(1.09-1.82)	0.009
Yes, <3	1.95(1.58-2.41)	<0.001	1.81(1.46-2.25)	<0.001	1.37(1.10-1.72)	0.006
Yes, ⩾3	3.22(2.63-3.95)	<0.001	2.85(2.32-3.51)	<0.001	1.98(1.58-2.48)	<0.001
Lung cancer						
No, <3	Ref.		Ref.		Ref.	
No, ⩾3	1.52(1.22-1.88)	<0.001	1.41(1.13-1.75)	0.002	1.39(1.12-1.73)	0.003
Yes, <3	1.76(1.42-2.17)	<0.001	1.68(1.36-2.08)	<0.001	1.41(1.13-1.76)	0.003
Yes, ⩾3	2.58(2.13-3.12)	<0.001	2.21(1.81-2.69)	<0.001	1.69(1.36-2.10)	<0.001
Digestive cancer*						
No, <3	Ref.		Ref.		Ref.	
No, ⩾3	1.09(0.87-1.36)	0.447	1.08(0.86-1.35)	0.503	1.02(0.82-1.28)	0.849
Yes, <3	1.61(1.33-1.96)	<0.001	1.50(1.23-1.84)	<0.001	1.27(1.03-1.57)	0.027
Yes, ⩾3	2.27(1.89-2.73)	<0.001	2.09(1.73-2.52)	<0.001	1.59(1.28-1.98)	<0.001
Colorectal cancer						
No, <3	Ref.		Ref.		Ref.	
No, ⩾3	1.29(0.97-1.72)	0.078	1.25(0.94-1.66)	0.126	1.22(0.91-1.63)	0.184
Yes, <3	1.37(1.02-1.85)	0.039	1.35(1.00-1.83)	0.051	1.00(0.73-1.36)	0.996
Yes, ⩾3	2.95(2.32-3.75)	<0.001	2.82(2.20-3.61)	<0.001	2.02(1.52-2.69)	<0.001
Others						
No, <3						
No, ⩾3	1.88(1.35-2.62)	<0.001	1.68(1.21-2.35)	0.002	1.60(1.15-2.24)	0.006
Yes, <3	2.06(1.49-2.85)	<0.001	2.06(1.48-2.86)	<0.001	1.38(0.96-1.97)	0.078
Yes, ⩾3	4.32(3.21-5.83)	<0.001	4.01(2.96-5.44)	<0.001	2.30(1.61-3.29)	<0.001
Tumor stage I						
No, <3	Ref.		Ref.		Ref.	
No, ⩾3	2.01(1.14-3.54)	0.016	1.99(1.12-3.54)	0.019	1.55(0.84-2.89)	0.164
Yes, <3	1.36(0.77-2.40)	0.283	1.11(0.63-1.98)	0.711	1.16(0.64-2.10)	0.635
Yes, ⩾3	3.13(1.86-5.27)	<0.001	2.52(1.48-4.29)	<0.001	1.78(0.94-3.39)	0.076
Tumor stage II						
No, <3	Ref.		Ref.		Ref.	
No, ⩾3	2.20(1.60-3.02)	<0.001	2.06(1.5-2.85)	<0.001	1.75(1.25-2.46)	0.001
Yes, <3	1.40(1.01-1.94)	0.043	1.24(0.89-1.72)	0.208	1.32(0.95-1.84)	0.102
Yes, ⩾3	3.03(2.25-4.08)	<0.001	2.51(1.84-3.41)	<0.001	2.42(1.71-3.44)	<0.001
Tumor stage III						
No, <3	Ref.		Ref.		Ref.	
No, ⩾3	1.44(1.13-1.83)	0.003	1.39(1.09-1.77)	0.007	1.17(0.91-1.51)	0.209
Yes, <3	1.37(1.08-1.74)	0.01	1.32(1.04-1.68)	0.023	1.27(0.99-1.62)	0.058
Yes, ⩾3	2.39(1.92-2.97)	<0.001	2.18(1.74-2.72)	<0.001	1.65(1.27-2.14)	<0.001
Tumor stage IV						

No, <3	Ref.		Ref.		Ref.	
No, ≥3	1.51(1.28-1.77)	<0.001	1.44(1.22-1.70)	<0.001	1.24(1.04-1.47)	0.016
Yes, <3	1.41(1.19-1.68)	<0.001	1.36(1.14-1.62)	<0.001	1.27(1.06-1.51)	0.008
Yes, ≥3	2.36(2.04-2.73)	<0.001	2.19(1.89-2.54)	<0.001	1.73(1.47-2.05)	<0.001
Radiotherapy, no						
No, <3	Ref.		Ref.		Ref.	
No, ≥3	1.81(1.59-2.07)	<0.001	1.73(1.52-1.98)	<0.001	1.3(1.13-1.49)	<0.001
Yes, <3	1.41(1.23-1.62)	<0.001	1.31(1.14-1.5)	<0.001	1.25(1.08-1.43)	0.002
Yes, ≥3	2.86(2.54-3.22)	<0.001	2.56(2.27-2.89)	<0.001	1.74(1.52-2.00)	<0.001
Radiotherapy, yes						
No, <3	Ref.		Ref.		Ref.	
No, ≥3	1.80(1.32-2.47)	<0.001	1.69(1.23-2.32)	0.001	1.2(0.86-1.68)	0.292
Yes, <3	1.54(1.13-2.08)	0.006	1.50(1.11-2.04)	0.009	1.33(0.98-1.82)	0.069
Yes, ≥3	3.51(2.69-4.58)	<0.001	3.04(2.31-4.00)	<0.001	1.96(1.42-2.70)	<0.001
Chemotherapy, no						
No, <3	Ref.		Ref.		Ref.	
No, ≥3	2.06(1.68-2.52)	<0.001	1.94(1.58-2.39)	<0.001	1.45(1.17-1.80)	<0.001
Yes, <3	1.41(1.15-1.73)	<0.001	1.31(1.07-1.61)	0.01	1.23(1.01-1.52)	0.044
Yes, ≥3	3.19(2.68-3.79)	<0.001	2.83(2.37-3.39)	<0.001	1.89(1.54-2.32)	<0.001
Chemotherapy, yes						
No, <3	Ref.		Ref.		Ref.	
No, ≥3	1.66(1.43-1.92)	<0.001	1.56(1.35-1.82)	<0.001	1.24(1.06-1.45)	0.008
Yes, <3	1.54(1.31-1.80)	<0.001	1.44(1.23-1.69)	<0.001	1.32(1.12-1.55)	<0.001
Yes, ≥3	2.96(2.57-3.40)	<0.001	2.62(2.27-3.01)	<0.001	1.78(1.52-2.08)	<0.001
PG-SGA, <4						
No, <3	Ref.		Ref.		Ref.	
No, ≥3	1.70(1.33-2.18)	<0.001	1.74(1.36-2.23)	<0.001	1.54(1.19-1.99)	<0.001
Yes, <3	1.52(1.24-1.86)	<0.001	1.40(1.14-1.71)	0.001	1.40(1.14-1.72)	<0.001
Yes, ≥3	1.81(1.36-2.43)	<0.001	1.82(1.36-2.44)	<0.001	1.38(1.02-1.88)	0.039
PG-SGA, ≥4						
No, <3	Ref.		Ref.		Ref.	
No, ≥3	1.66(1.43-1.91)	<0.001	1.60(1.38-1.85)	<0.001	1.25(1.08-1.45)	0.004
Yes, <3	1.33(1.13-1.56)	<0.001	1.27(1.08-1.49)	0.001	1.22(1.04-1.43)	0.017
Yes, ≥3	2.75(2.41-3.13)	<0.001	2.51(2.20-2.87)	<0.001	1.81(1.57-2.09)	<0.001

Data presented as hazard ratio (95% CI).

[a] Cox proportional hazards models without adjust;

[b] Cox proportional hazards models adjust for age, gender, body mass index;

[c] Cox proportional hazards models adjust for age, gender, body mass index, smoking, alcohol, tumor location, tumor stage, chemotherapy, radiotherapy, surgery, nutrition intervention, ECOG, PG-SGA, Place of Residence, Educational attainment, hand grip strength, EORTC QLQ-C30.

Digestive cancer* : Digestive cancer except colorectal cancer

Supplementary table 8. Sensitive analysis.

Frailty and NLR	ECOG≤1		Excluding patients dying within 6 months		Excluding other affected factors*	
	HR(95%CI)	P-value	HR(95%CI)	P-value	HR(95%CI)	P-value
No, <3	ref		ref		Ref	
No, ⩾3	1.29 (1.12,1.48)	< 0.001	1.12 (0.97,1.30)	0.132	1.21(1.11,1.59)	<0.001
Yes, <3	1.29 (1.13,1.47)	< 0.001	1.33 (1.15,1.54)	< 0.001	1.30(1.12,1.64)	<0.001
Yes, ⩾3	1.72 (1.49,1.98)	< 0.001	1.66 (1.43,1.92)	< 0.001	1.79(1.31,2.01)	<0.001

Cox proportional hazards models adjust for age, gender, body mass index, smoking, alcohol, tumor location, tumor stage, chemotherapy, radiotherapy, surgery, nutrition intervention, ECOG, PG-SGA, Place of Residence, Educational attainment, hand grip strength, EORTC QLQ-C30.

*Excluding perioperative patients, patients with blood tumors and chemotherapy.

Supplementary table 9. Neutrophil to Lymphocyte Ratio (NLR), Frailty, and 30 days mortality

**Frailty and NLR**	**30 days mortality**	
	**OR(95%CI)**	***P*-value**
No, <3	ref	
No, ⩾3	0.96 (0.38,2.44)	0.938
Yes, <3	1.49 (0.60,3.71)	0.395
Yes, ⩾3	2.80 (1.24,6.29)	0.013

Cox proportional hazards models adjust for age, gender, body mass index, smoking, alcohol, tumor location, tumor stage, chemotherapy, radiotherapy, surgery, nutrition intervention, ECOG, PG-SGA, Place of Residence, Educational attainment, hand grip strength, EORTC QLQ-C30.

Supplementary table 10. Estimated Value of Four Classification Models in Predicting Mortality

**Model**	**C-index**	***P*-value**
TNM stage	0.655(0.644-0.666)	Ref
TNM stage + NLR	0.684(0.672-0.696)	0.403†
TNM stage + frailty	0.687(0.676-0.698)	<0.001†
TNM stage + NLR+ frailty	0.692(0.680-0.703)	<0.001†

† Compared with TNM stage.

Cox models were adjusted for age, gender, body mass index, smoking, alcohol, tumor location, tumor stage, chemotherapy, radiotherapy, surgery, nutrition intervention, ECOG, PG-SGA, Place of Residence, Educational attainment, hand grip strength, EORTC QLQ-C30.

Supplementary Figure 1. Flow chart

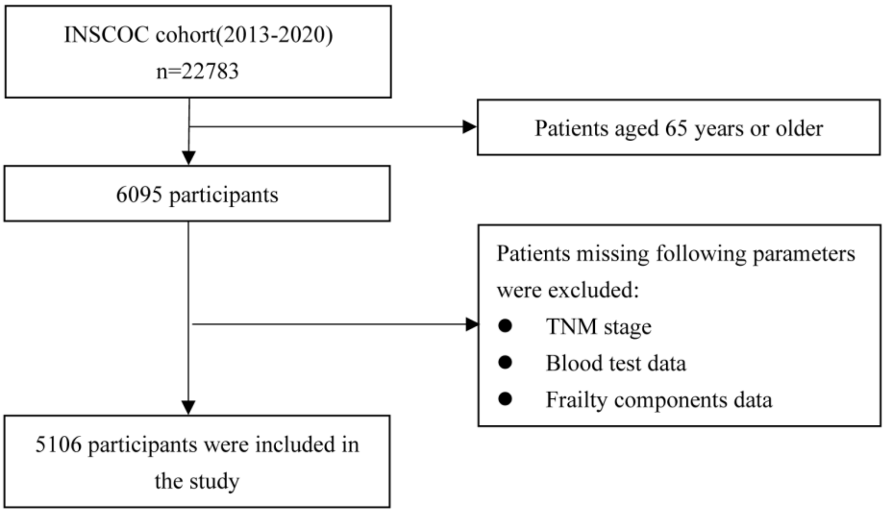

Supplementary Figure 2. Kaplan-Meier curves according to frailty and Neutrophil to Lymphocyte Ratio in elderly patients with cancer, respectively.

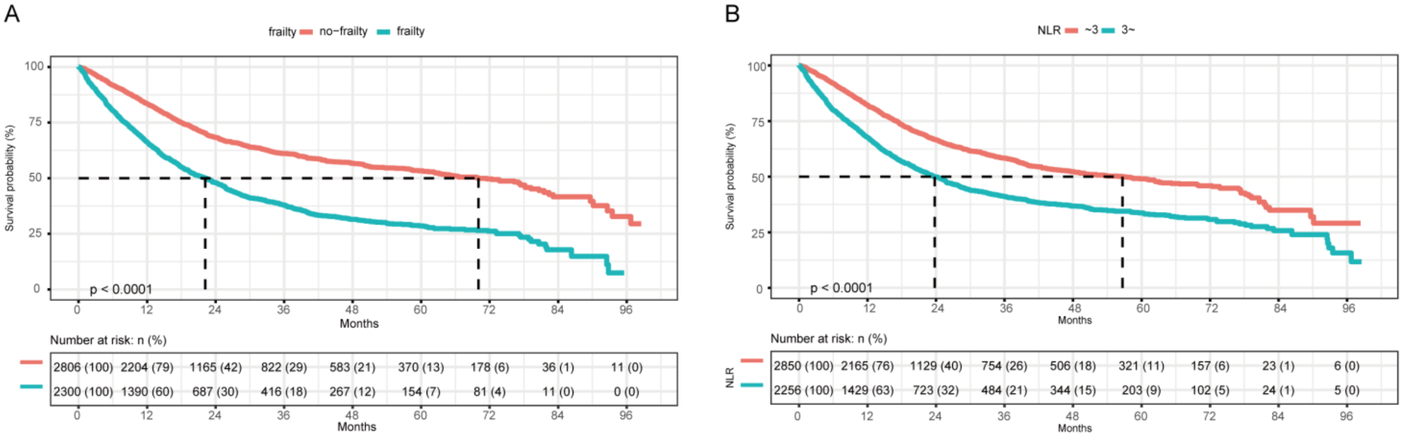

Supplementary Figure 3. Age-Distribution according to number of frailty risk factors, Neutrophil to Lymphocyte Ratio and frailty in elderly patients with cancer.

A B

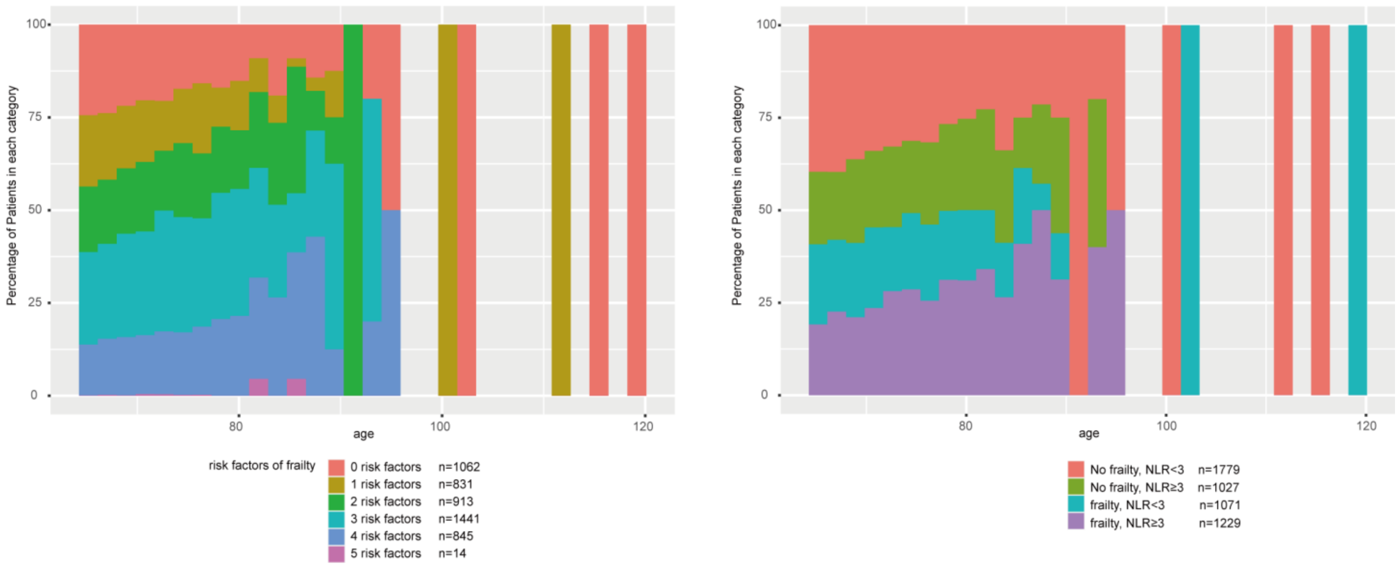

Supplementary Figure 4. Cumulative incidence curves according to frailty and Neutrophil to Lymphocyte Ratio in elderly patients with cancer, respectively.

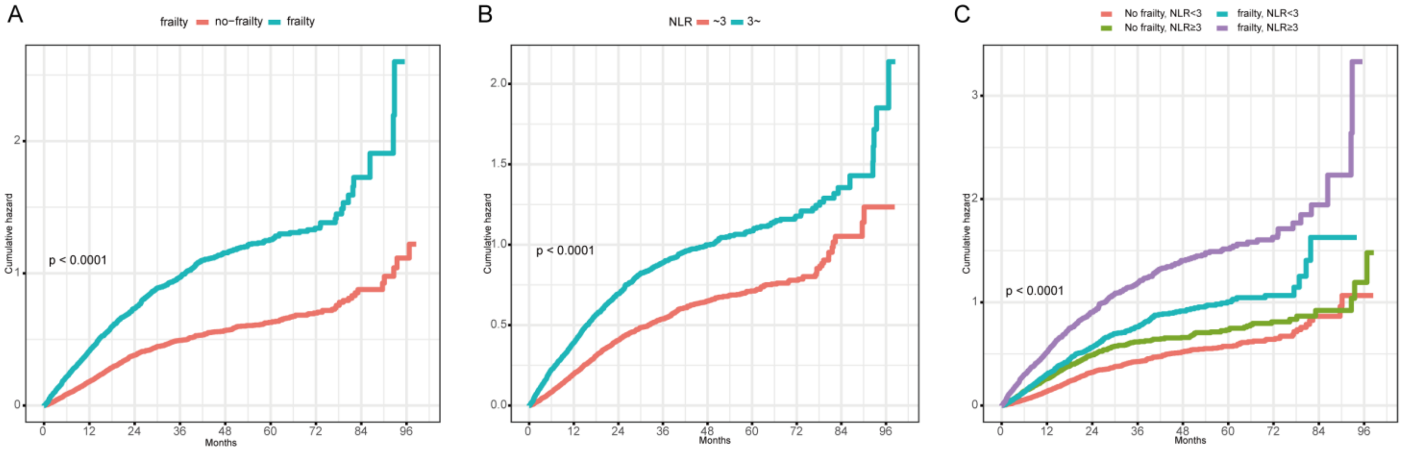

Supplementary Figure 5. Kaplan-Meier curve According to Neutrophil to Lymphocyte Ratio and frailty present in patients receiving nutritional intervention.

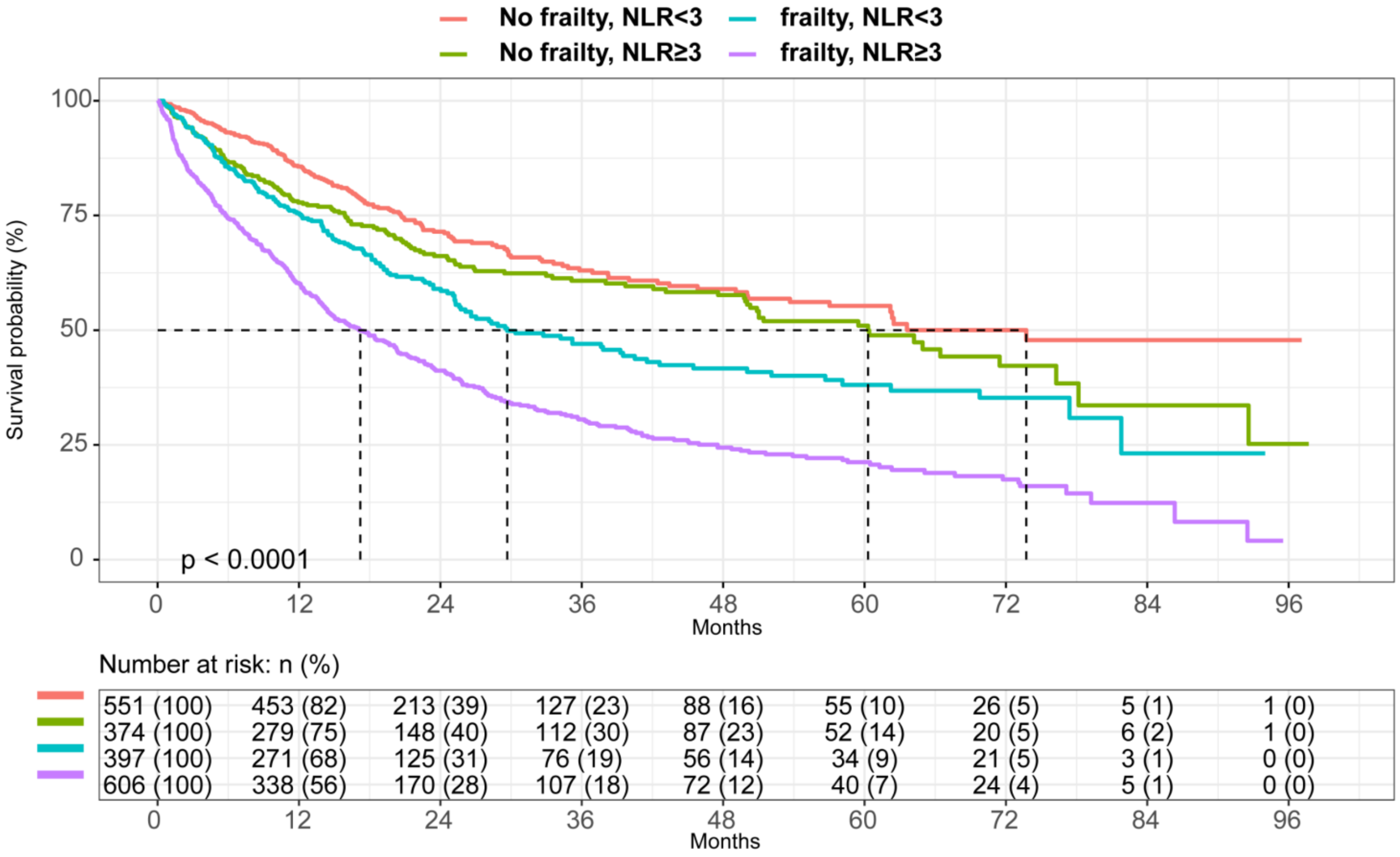

Supplementary Figure 6. Kaplan-Meier curves according to components of frailty.

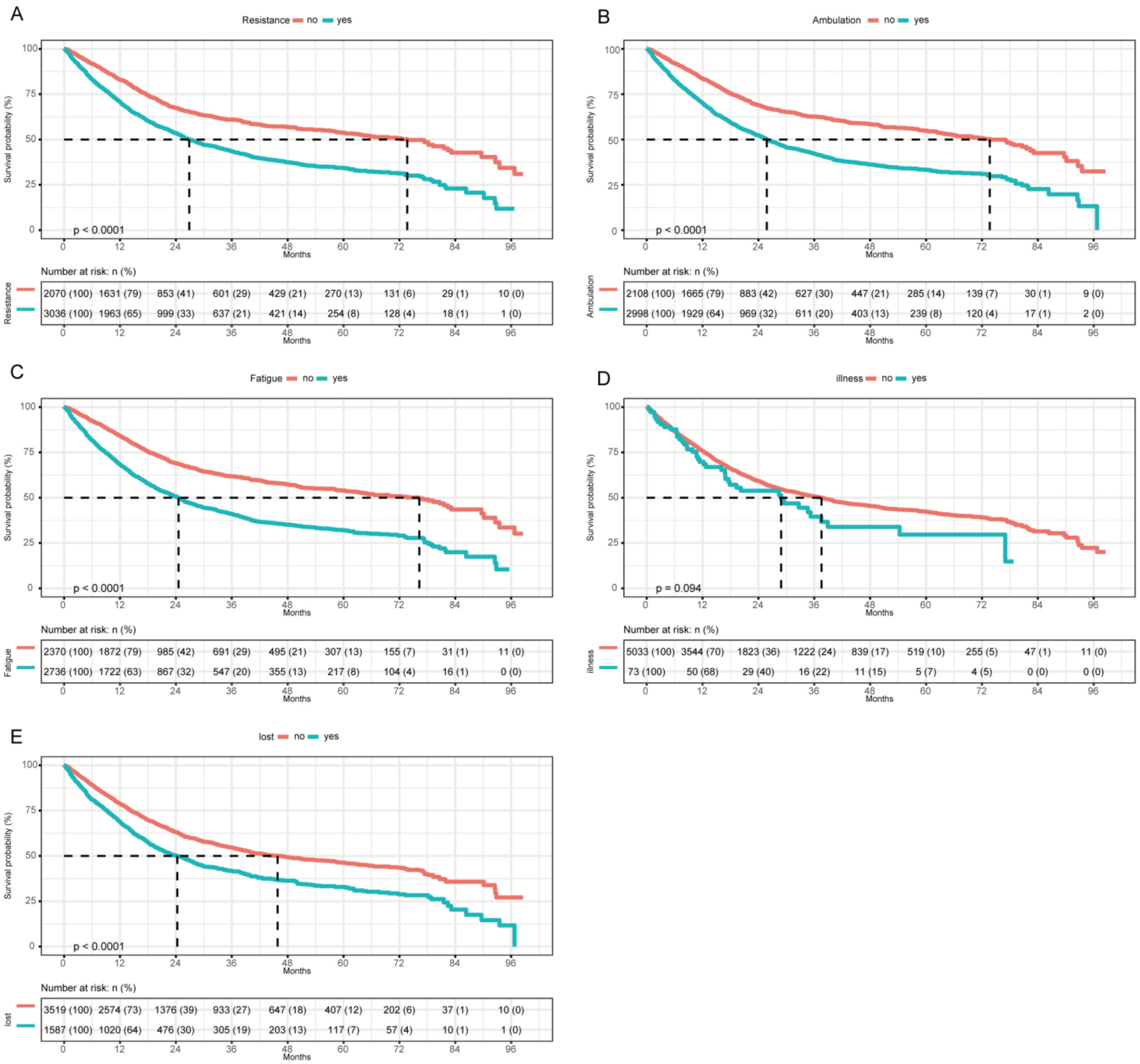

Supplementary Figure 7. Kaplan-Meier curves according to number of risk factors of frailty.

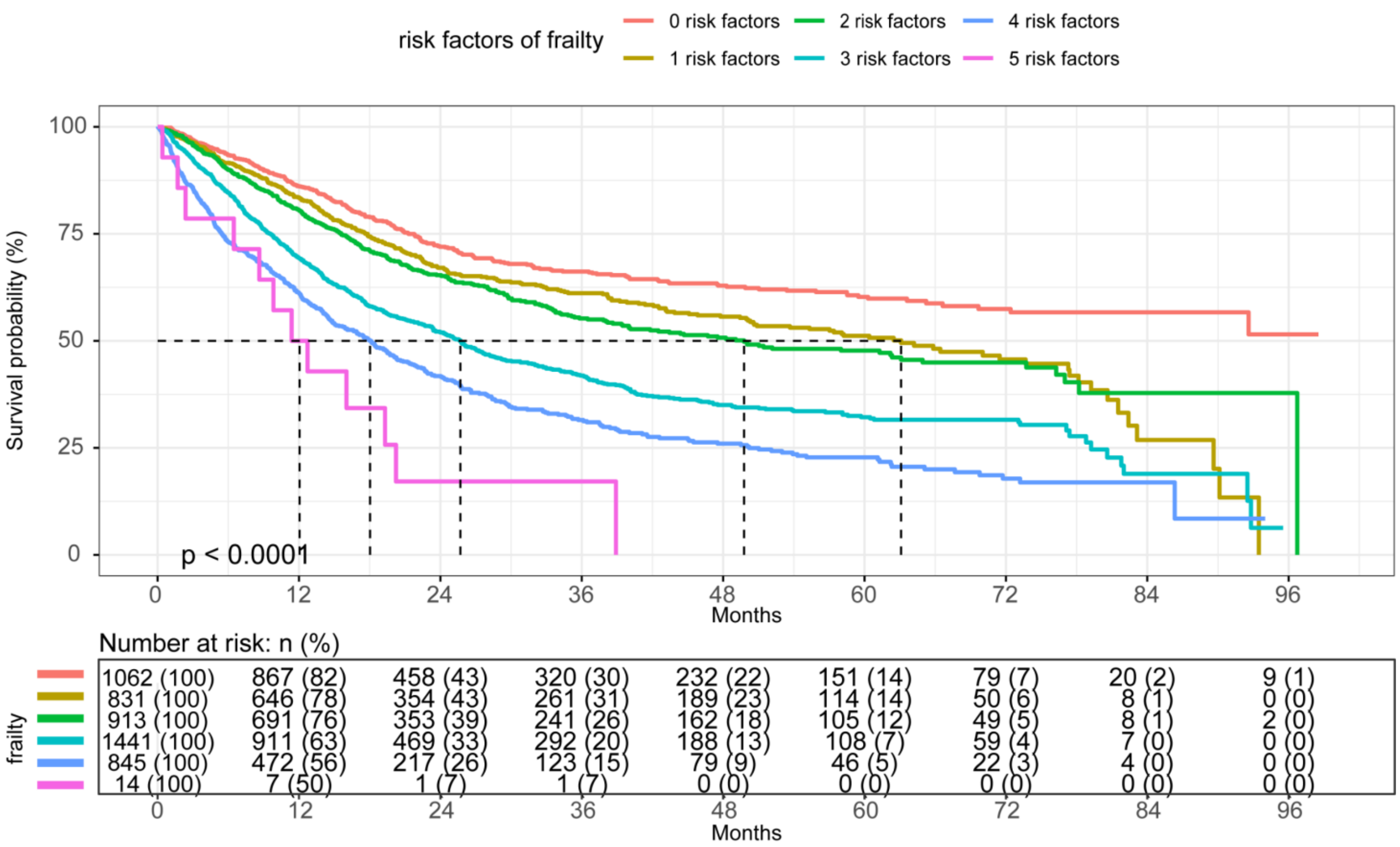

Supplement: Supplementary file 1 [file DataSheet_1.pdf]
